# Dxr is essential in *Mycobacterium tuberculosis *and fosmidomycin resistance is due to a lack of uptake

**DOI:** 10.1186/1471-2180-8-78

**Published:** 2008-05-20

**Authors:** Amanda C Brown, Tanya Parish

**Affiliations:** 1 Institute for Cell and Molecular Science, Barts and the London School of Medicine and Dentistry, London, UK

## Abstract

Fosmidomycin is a phosphonic antibiotic which inhibits 1-deoxy-D-xylulose 5-phosphate reductoisomerase (Dxr), the first committed step of the non-mevalonate pathway of isoprenoid biosynthesis. In *Mycobacterium tuberculosis *Dxr is encoded by Rv2870c, and although the antibiotic has been shown to inhibit the recombinant enzyme [1], mycobacteria are intrinsically resistant to fosmidomycin at the whole cell level. Fosmidomycin is a hydrophilic molecule and in many bacteria its uptake is an active process involving a cAMP dependent glycerol-3-phosphate transporter (GlpT). The fact that there is no *glpT *homologue in the *M. tuberculosis *genome and the highly impervious nature of the hydrophobic mycobacterial cell wall suggests that resistance may be due to a lack of cellular penetration.

**Results:**

We demonstrated that *dxr *(Rv2780c) is an essential gene in *M. tuberculosis*, since we could not delete the chromosomal copy unless a second functional copy was provided on an integrating vector. This confirmed that the intracellular target of fosmidomycin was essential as well as sensitive. We looked at the uptake of fosmidomycin in two mycobacterial species, the slow-growing pathogenic *M. tuberculosis *and the fast-growing, saprophytic *Mycobacterium smegmatis*; both species were resistant to fosmidomycin to a high level. Fosmidomycin was not accumulated intra-cellularly in *M. tuberculosis *or *M. smegmatis *but remained in the extra-cellular medium. In contrast, fosmidomycin uptake was confirmed in the sensitive organism, *Escherichia coli*. We established that the lack of intra-cellular accumulation was not due to efflux, since efflux pump inhibitors had no effect on fosmidomycin resistance. Finally, we demonstrated that fosmidomycin was not modified by mycobacterial cells or by extracts but remained in a fully functional state.

**Conclusion:**

Taken together, these data demonstrate that fosmidomycin resistance in M. tuberculosis and M. smegmatis results from a lack of penetration of the antibiotic to the site of the sensitive target.

## Background

The mycobacteria contain a number of important pathogens which infect both animals and humans. The World Health Organisation (WHO) has estimated that eight million humans per annum are newly infected with *Mycobacterium tuberculosis*, (the main causative agent of human tuberculosis), resulting in almost two million deaths per year [2]. Currently the only available vaccine is the live, attenuated *Mycobacterium bovis *Calmette-Guerin (BCG) strain; however, BCG does not offer complete immunity, and protection is highly variable due to a wide range of social, economic and environmental factors. Opportunistic mycobacterial pathogens have been identified as causing disseminated disease in HIV-infected or otherwise immuno-compromised individuals. In addition a number of other serious diseases, including leprosy and Buruli ulcer, are caused by mycobacteria. Many of these infections are on the increase and, although effective therapy exists for some of these diseases, an increase in multi-drug resistance strains jeopardises our ability to treat them. Current research trends are focused on the production of an improved vaccine, identification of new drug targets, and the development of new anti-mycobacterials. All of these activities have benefited greatly from the availability of the complete genome sequence of *M. tuberculosis *[3].

The need for new antibiotics effective against the mycobacteria has never been greater. In addition to a search for new antibiotics, there has been renewed interest in examining existing compounds for efficacy as anti-mycobacterial agents. Mycobacteria are relatively antibiotic resistant and are not susceptible to many commonly used antibiotic groups, such as the penicillins. This intrinsic resistance has been attributed largely to the nature of the mycobacterial cell wall, which is rich in long-chain fatty acids including the C_60 _to C_90 _mycolic acids, which are covalently linked to the arabinogalactan-peptidogylcan layer. Porins, (water-rich channel proteins which allow hydrophilic molecules to enter the cell via diffusion), are rare in mycobacteria [4] and have been found to function at a considerably reduced rate in comparison to porins in Gram-negative bacteria in *Mycobacterium smegmatis *[5-7]. Therefore antibiotic resistance is often due to the physical properties of the cell wall forming an impermeable barrier [8], rather than drug inactivation and it has been assumed that the bacteria have susceptible intracellular targets, so that if the drugs were modified to allow cellular entry they would become effective.

Isopentenyl disphosphate (IPP) is a common precursor in the biosynthesis of all isoprenoid compounds. This includes polyprenyl phosphate, which is involved in the synthesis of the covalently linked peptidoglycan-arabinogalactan-mycolic acid complex, lipomannan and lipoarabinomannan [9]. Isoprenoids can be synthesized by two pathways; the mevalonate pathway (which is present in humans), and the non-mevalonate or 1-deoxy-D-xylulose 5-phosphate (DOXP) pathway, which has been found in many bacteria and parasites. Genome sequencing of *M. tuberculosis *has shown that the non-mevalonate pathway is the sole pathway present [3]. Since this pathway is absent from humans it represents an attractive target for drug development.

Fosmidomycin (sodium hydrogen 3-(*N*-hydroxyfomamido)propylphosphonate) is a phosphonate antibiotic which inhibits one of the enzymes of the DOXP pathway – the DOXP reductoisomerase (Dxr) [10]. However, antimicrobial activity has been shown to be limited to Gram-negative species, and fosmidomycin is not effective against Gram-positive cocci or anaerobic species [11,12]. In *Escherichia coli*, fosmidomycin is transported into the cell via the glycerol-3-phosphate transporter, GlpT, and *glpT *deletion mutants are fosmidomycin resistant [13]. A fosmidomycin resistance gene (*fsr*) has also been identified in several bacteria including *E. coli *and *Brucella *spp. [14,15]. Fsr is a member of a family of permeases, suggesting that resistance results from rapid efflux of the drug from the cell. Fosmidomycin is currently being considered as a treatment for malaria [16-18].

Mycobacteria, including *M. tuberculosis*, are highly resistant to fosmidomycin at the whole cell level, although recombinant *M. tuberculosis *Dxr is sensitive to fosmidomycin, and the mechanism of resistance is unknown [1]. In this study, we characterized the basis of the intrinsic resistance of *M. tuberculosis *and *M. smegmatis *to fosmidomycin. We demonstrated that the *dxr *gene is essential in *M. tuberculosis*, confirming that the organism has a sensitive and vulnerable target. We investigated uptake of fosmidomycin into mycobacteria and *E. coli *using a bioassay for fosmidomycin. Efflux pump inhibitors were used to assess the role of efflux in mediating fosmidomycin resistance. Inactivation of fosmidomycin by mycobacterial cells or cell-free extracts was also tested.

## Results

### Essentiality of Dxr

We were interested in determining the basis of mycobacterial resistance to fosmidomycin. The target of fosmidomycin is the enzyme Dxr (DOXP reductoisomerase, Rv2870c), the first committed step in the non-mevalonate pathway of isoprenoid biosynthesis. *M. tuberculosis *Dxr has been expressed as a recombinant protein and is readily inhibited by fosmidomycin [1], indicating that resistance is not due to lack of a susceptible target. One possibility to account for the lack of whole cell sensitivity is that Dxr is not required for mycobacterial growth. The non-mevalonate pathway is the only known pathway of isoprenoid biosynthesis in mycobacteria [19], so it seems unlikely that this is the case. However, work using saturating transposon mutagenesis [20] gave rise to the prediction that *dxr *is not an essential gene.

In order to address this question, we used a two-step homologous recombination method to attempt to generate a defined mutant of *dxr *in *M. tuberculosis *[21]. A deletion delivery vector, pORTAL3- with a defined deletion of *dxr*, was introduced into *M. tuberculosis *and single cross overs (SCOs) were obtained. Double cross-overs (DCOs) were then generated from the SCOs and screened by PCR to determine if the wild type or the deletion allele was present. Of the 40 DCOs screened all were found to be wild type, suggesting essentiality of *dxr*.

**Figure 1:**
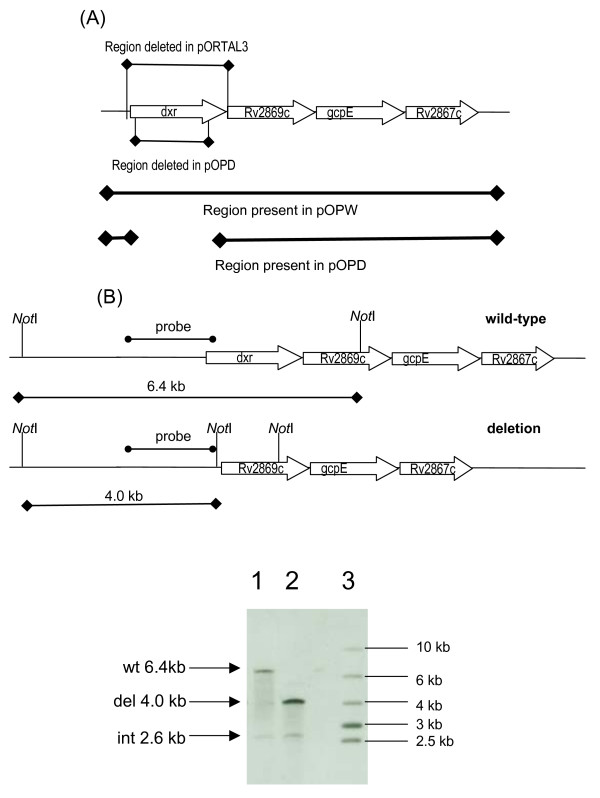
**Demonstration of the essentiality of *dxr***. (A) The chromosomal location of the *dxr *operon, the region deleted in the delivery vector (pORTAL3), and the regions present in the complementing vectors pOPW (complete operon) and pOPD (operon without *dxr*) are shown. (B) The deletion delivery vector pORTAL3 was introduced into the chromosome by homologous recombination followed by site-specific recombination with the complementing vector pOPW to generate a merodiploid strain. DCOs were generated in this background and analysed. Southern analysis of representative DCOs isolated in the merodiploid background is shown. Genomic DNA was digested with *Not*I and hybridised to the indicated probe. The sizes for the wild-type and deletion alleles are shown diagrammatically – replacement of the wild-type gene results in an additional *Not*I site in the chromosome. Lane 1 – wild-type DCO (*dxr*^*wt*^, *dxr*^int^), Lane 2 – Deletion DCO (*dxrΔ*, *dxr*^int^), Lane 3 – markers. The bands for the wild-type, deletion and integrated copies of *dxr *are indicated.

Since we were unable to isolate a deletion mutant in the wild type background, we constructed a merodiploid strain carrying the entire *dxr *operon with its native promoter on a mycobacteriophage L5 based integrating vector (pOPW: *dxr*^*int*^*gm*). DCOs were generated in this background as before and screened by PCR. Of the 24 DCOs screened 12 had the deletion allele (50%), indicating that we were able to isolate chromosomal deletion of *dxr *in this background. The expected genotype of a number of these strains, which we have termed "del-inquents" i.e. a deleted copy on the chromosome and an integrated, functional gene was confirmed by Southern analysis (Figure 1B). This data confirms that the *dxr *operon is essential (since we complemented with the whole operon).

Since *dxr *is in an operon (Figure 1A), it is possible that the inability to isolate mutants could be due to one of the downstream genes being disrupted in the mutant. Analysis of the mutation that would be created by the pORTAL3 delivery vector suggests that it may disrupt the promoter region at the start of the operon and also the gene downstream of *dxr *(Rv2869c), a non-essential intramembrane-cleaving protease (iCLIP) [22,23].

In order to confirm that *dxr *alone is essential, we used a gene switching strategy. This relies on the fact that efficient replacement of a resident integrated vector can easily be achieved in *M. tuberculosis *by transformation and selection for a second integrating plasmid carrying a different antibiotic resistance marker [24-26]. We made use of the delinquent strain carrying the complete operon (pOPW) (*dxr*Δ, *dxr*^*int *^*gm*) and tested the ability of a plasmid carrying the whole operon except *dxr *(pOPD; Figure 1A) to complement the chromosomal mutation. The delinquent strain was transformed with pOPD and plated onto hygromycin plates to select for the incoming vector. No hygromycin resistant transformants were obtained, although a control vector transformed the same cells at an efficiency of 1.4 × 10^6^per μg. In contrast replacement of pOPW by pOPD in a wild-type background was easily achieved at a efficiency of 1.2 × 10^6 ^per μg. These data indicate that a vector lacking only *dxr *does not complement the chromosomal mutation, and therefore that *dxr *itself is essential. It is still possible that other genes in the operon are essential as well and this strategy could in the future be extended to examining the essentiality of other genes in this operon.

### Resistance to fosmidomycin is not due to efflux

**Figure 2:**
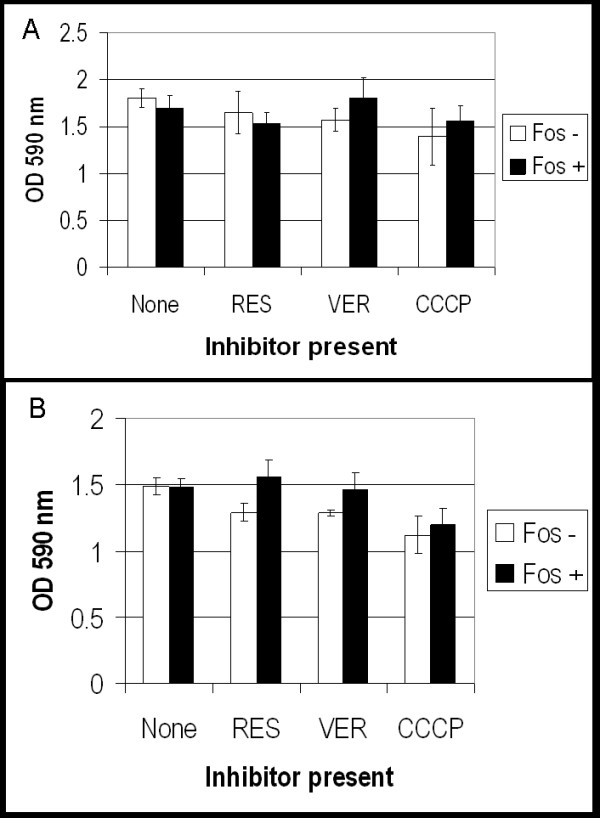
**Efflux pump inhibitors have no effect on mycobacterial resistance to fosmidomycin**. Mycobacteria were grown in the presence of 100 μg/ml fosmidomycin (Fos), 12 μg/ml reserpine (RES), 40 μg/ml verapamil (VER) and 1.25 μg/ml carbonyl cyanide *m*-chlorophenylhydrazone (CCCP) where indicated. (A) *M. tuberculosis *was grown for 28 d and (B) *M. smegmatis *for 16 h. Error bars represent standard deviation of triplicate samples.

We confirmed that mycobacteria were highly resistant to fosmidomycin resistance in our culture conditions. We looked at the growth of *M. smegmatis *and *M. tuberculosis *in liquid medium in the presence of fosmidomycin. No inhibition of growth was seen at any concentration up to 1 mg/ml (Figure 2A & B), confirming that both species are resistant to a high level. Since *dxr *is essential in normal culture conditions and that Dxr is sensitive to fosmidomycin inhibition [1], there must be another explanation for the high level resistance.

Resistance to fosmidomycin has been attributed to the Fsr efflux pump in several micro-organisms [14,15]. Mycobacteria are known to posses many efflux pumps which confer intrinsic antibiotic resistance [27-32]. We investigated whether efflux plays a role in fosmidomycin resistance in mycobacteria, by utilising the efflux pump inhibitors, reserpine, verapamil and carbonyl cyanide *m*-chlorophenylhydrazone (CCCP). The concentrations of efflux pump inhibitors we used were taken from a previous study in *M. smegmatis *[27]. As before, reserpine and verapamil at 12 and 40 μg/ml respectively were not inhibitory to growth. However, CCCP at a concentration of 15 μg/ml was seen to significantly inhibit growth in both *M. smegmatis *and *M. tuberculosis*. Therefore, we tested lower concentrations of CCCP; 1.25 μg/ml was the highest permitted concentration where growth rate was not notably affected, although some inhibition was still seen.

Growth of mycobacteria in the presence of efflux pump inhibitors and fosmidomycin was assayed. All cultures grew to an OD of above 1.0 (approx 10^9 ^cell per ml), indicating that there were no significant effects on growth rate or the final OD reached. No difference in the sensitivity of either species to fosmidomycin was seen in the presence of the inhibitors, indicating that efflux is not the mechanism of resistance.

### Transport of fosmidomycin into cells

Since we had discounted efflux as the resistance mechanism, we determined whether fosmidomycin was transported into the mycobacterial cells in the first place. Uptake of antibiotics is typically measured using a radiolabelled derivative. However, radio-labelled fosmidomycin is not available commercially and there are additional associated hazards connected with using radio-isotopes under Category Three level containment in the UK. A microbiological bioassay, based on the inhibition of *E. coli *by active compounds, has previously been used to measure fosmidomycin concentrations [11,33]. In this assay, active fosmidomycin is measured by inhibition of *E. coli *growth on disks. A standard curve using known concentrations of fosmidomycin can be generated and used to quantify fosmidomycin [see Additional file 1] [34].

**Figure 3:**
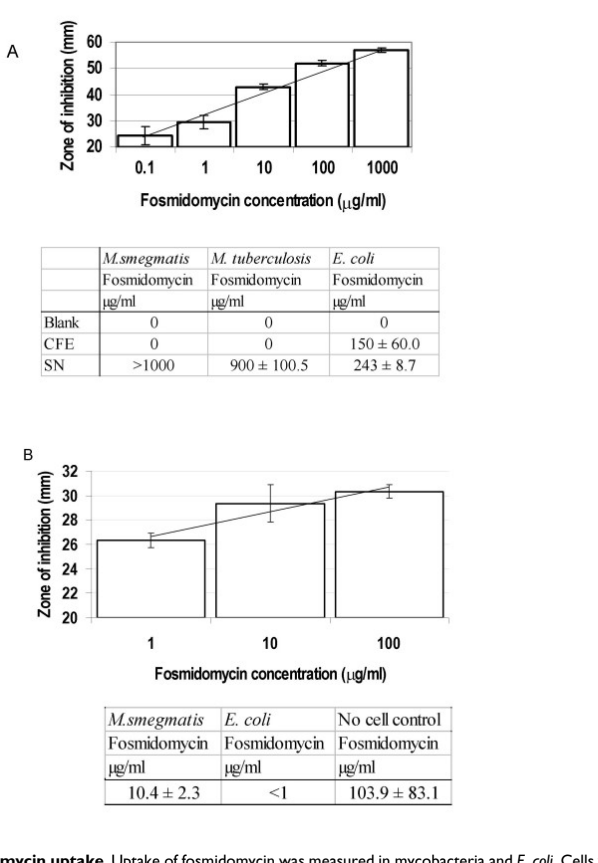
**Assay of fosmidomycin uptake**. Uptake of fosmidomycin was measured in mycobacteria and *E. coli*. Cells were subjected to (A) 1 mg/ml of fosmidomycin for 30 min; (B) 20 μg/ml of fosmidomycin for 60 min. Cell-free extracts (CFE) or culture supernatants (SN) were prepared and serial dilutions were applied to paper disks as described in the methods. For each assay, a standard curve was generated using known concentrations of fosmidomycin. For test samples, zones of inhibition against *E. coli *were measured and the antibiotic concentration calculated from the standard curve.

*M. smegmatis, M. tuberculosis *and *E. coli *cells were incubated with 1 mg/ml of fosmidomycin for 30 min (Figure 3A). Active fosmidomycin in the supernatant (extra-cellular) and cell-free extracts (intra-cellular) was measured. No active fosmidomycin was present in the cell-free extracts generated from *M. tuberculosis *or *M. smegmatis*; all of the fosmidomycin remained in the supernatant. In contrast, *E. coli *extracts contained more than 150 μg/ml and a reduction in the amount of fosmidomycin in the supernatant was seen. These data confirmed fosmidomycin uptake in *E. coli*, and conversely that there is a lack of uptake by mycobacteria.

The limit of detection for uptake in this assay was 0.1 μg/ml, which is 10,000-fold less than the level of antibiotic in the medium. One of the limitations of using a high concentration of fosmidomycin with *E. coli *is that some of the antibiotic in the supernatant could have been released from the cells after antibiotic-induced lysis. However, this would lead us to underestimate uptake, rather than overestimate it.

### Detoxification/modification

The results from the transport assay strongly suggested that fosmidomycin resistance in mycobacteria results from a lack of active transport. However, it was not possible to tell if the antibiotic was being modified in the previous assay. This was because a high concentration of antibiotic was used; if only a small proportion of the fosmidomycin were modified, it would not have been detected as a loss of fosmidomycin activity in the supernatant. Thus, the possibility that the cells might be modifying the antibiotic into a non-toxic form still existed. To address this question we repeated the exposure assay using a lower concentration of fosmidomycin which allowed more precise quantification of the active antibiotic in the supernatant. *M. smegmatis *or *E. coli *cells were incubated with 20 μg/ml of fosmidomycin for 60 min and active fosmidomycin assayed in the supernatant (Figure 3B). In the supernatant exposed to *M. smegmatis*, a 2-fold reduction in activity (from 20 to 10 μg/ml) was seen. Given the limitation of the assay, where such a difference would result in a change in the zone of inhibition of less than 1 mm, this is likely to be due to lack of sensitivity in the assay. In addition, if detoxification were occurring we would expect to see a complete reduction of fosmidomycin activity. In contrast a 100-fold reduction in fosmidomycin activity was seen in the *E. coli *supernatants (due to uptake from the extra-cellular environment into the cell). Thus, no modification of the antibiotic by *M. smegmatis *was seen.

**Figure 4:**
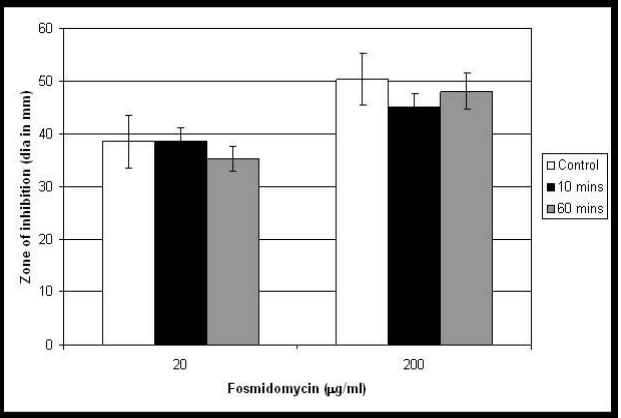
**Assay of fosmidomycin detoxification by cell free extracts**. Cell free extracts (CFE) were tested for inactivation of fosmidomycin. Fosmidomycin, at a final assay concentration of 20 or 200 μg/ml, was incubated with sterile 10 mM Tris pH8 (control), or *M. smegmatis *cell-free extract for 10 or 60 min. A 100 μl aliquot from each test sample was applied to a sterile paper disc and aseptically placed on media containing *E. coli*. The diameter of the zone of growth inhibition was measured. Error bars are the standard deviation of triplicate samples.

Although fosmidomycin is not taken up into the mycobacterial cells, there could still be intra-cellular enzymes capable of detoxification. In order to address this, we determined whether extracts of *M. smegmatis *were capable of inactivating fosmidomycin. *M. smegmatis *cell-free extracts were incubated with fosmidomycin (20 μg/ml and 200 μg/ml) for 10 to 60 minutes. No decrease in fosmidomycin activity was seen during this time, indicating that no detoxifying enzymes are present (Figure 4). These data confirmed that resistance is not due to modification of fosmidomycin.

## Discussion

Using a two-step recombination process, we have demonstrated that Rv2870c (*dxr*) is essential for growth in *M. tuberculosis in vitro*. This is contradictory to previous reports from TraSH analysis, which predicted *dxr *to be a non-essential gene in *M. tuberculosis *[20]. In the TraSH system predictions of gene essentiality are based on a ratio generated from microarray analysis of a transposon library, with an arbitrary cut-off point used to distinguish essential and non-essential genes. A closer inspection of the data for *dxr *suggests that its prediction as non-essential may be incorrect, since the ratio obtained is very close to the cut-off point. Our data demonstrate that this is indeed the case, since we were only able to construct a chromosomal deletion of *dxr *in a merodiploid background. However, although we were able to demonstrate that *dxr *is essential for growth *in vitro*, before it can be truly validated as a drug target it needs to be proved essential *in vivo*. Whilst this is extremely challenging [35], the use of inducible systems for generating conditional strains, as recently described, may now allow this to be carried out [36-38].

## Conclusion

Our data demonstrate that fosmidomycin is not taken up by mycobacteria, and resistance results from the intra-cellular target being inaccessible to the drug. Since, we have validated *dxr *as an essential gene, it is still possible that it could prove to be a useful drug target if inhibitors which enter the cell are developed. Alternatively fosmidomycin could be used in conjunction with a compound which increases permeability. Other approaches could involve nano-particulation of fosmidomycin or the conversion of fosmidomycin into a pro-drug, which would be readily taken up by the cell and then hydrolysed into an active form once inside the cell structure.

## Methods

### Culture of mycobacteria

*M. tuberculosis *H37Rv was cultured in Middlebrook 7H9 liquid medium supplemented with 10% v/v OADC (oleic acid, bovine serum albumin, D-glucose, catalase; Becton Dickinson) and 0.05 % w/v Tween 80 (7H9/Tw/OADC) or on solid Middlebrook 7H10 agar supplemented with 10% v/v OADC (7H10/OADC). *M. smegmatis *mc^2^155 was grown on Lemco media [39]. X-Gal (5-bromo-4-chloro-3-indolyl-β-D-galactopyranoside) was used at 50 μg/ml; IPTG (isopropyl-beta-D-thiogalactopyranoside) at 0.5 mM; kanamycin at 20 μg/ml; hygromycin B at 100 μg/ml; and sucrose at 2% w/v. Fosmidomycin (sodium salt) was purchased from Invitrogen. Efflux pump inhibitors: reserpine was used at 12 μg/ml; verapamil at 40 μg/ml; and carbonyl cyanide *m*-chlorophenylhydrazone (CCCP) at 1.25 μg/ml. *M. tuberculosis *growth curves were conducted in 4 ml of 7H9/Tw/OADC media in 16 mm glass tubes containing an 8 mm magnetic stirrer bar, stirring at 150 rpm.

### Attempt to construct deletion mutants of dxr

A deletion delivery vector for *dxr *(pORTAL3) was constructed as follows (Figure 1): the upstream flanking region was amplified from *M. tuberculosis *genomic DNA using the primer pair DXR US F (*Pst*I) 5'-TGGGCTGCAGCAACCCGCTAAGAAC-3, and DXR US Rev (*Not*I) 5'-CCCGCGGCCGCTTGATGCTAAGATGCCATGC-3' and the downstream flanking region was amplified using the primer pair DXR DS F (*Not*I) 5'-CCCGCGGCCGCTGTTTGTTACCGGCATTGTG-3' and DXR DS Rev (*Hin*dIII) 5'-CCCAAGCTTGGGCCAAGAAGAACCAGAAC-3'. Fragments were cloned into p2NIL (*Pst*I-*Hin*dIII) using the underlined restriction sites. The 6.3 kb *Pac*I cassette from pGOAL19 (*hyg, sacB, lacZ*) was then cloned into the sole *Pac*I site. The vector was verified by restriction digest and sequencing. 5 μg of UV pre-treated plasmid DNA [40] was electroporated into competent *M. tuberculosis *and SCO transformants were selected on 7H10/OADC medium containing kanamycin and hygromycin. DCOs were isolated by streaking cells onto plates lacking antibiotics and selected/screened for on media containing sucrose and X-gal as previously described [21]. PCR screening on DCOs was carried out using primers DXR OL F (5'-TTGGACGATAGATCGACACC-3') and DXR OL Rev (5'-GATGGCATAGATCAGCACCA-3').

A complementing vector carrying the entire *dxr *operon with its native promoter was constructed by amplifying a 4.7 kb region from genomic H37Rv DNA with primers DXR OP C' F 5'-TTAATTAACACCTCGCGGTACAGCTCGTC-3' and DXR OP C' Rev 5'-TTAATTAAGTCGTCAGCGCTGATATGCCC-3'. The product was cloned into pSC-A (Stratagene); to give dxr_op/pSCA, and the Gm-int cassette from pUC-Gm-Int [41] was introduced as a *Hin*dIII fragment to give pOPW (Figure 1).

### Construction of merodiploid strain and confirmation of essentiality

We constructed a merodiploid strain by electroporating the *dxr *SCO with pOPW and isolating kanamycin/hygromycin/gentamicin resistant transformants. DCOs were isolated and PCR-screened as before. Strains were confirmed by Southern analysis using the AlkPhos Direct system (GE Healthcare) to label and detect DNA. A deletion derivative of dxr_op/pSCA was constructed in which the 1.2 kb *dxr *gene was removed by site directed mutagenesis with primers DXR KO SDM F 5'-GTGACCAACTCGACCGACGTATCTGGTATGGCTTCG-3' and DXR KO SDM Rev 5'-CGAAGCCATACCAGATACGTCGGTCGAGTTGGTCAC-3'. The Hyg-Int cassette from pUC-Hyg-Int [41] was cloned in via a *Hin*dIII digest. The resulting plasmid, pOPD (Figure 1), was used in a gene switching experiment [25]. Strains were electroporated with integrating vectors and transformants isolated on the appropriate antibiotic selection (for the incoming vector). Transformants were patch tested for antibiotic resistance as required.

### Fosmidomycin uptake assays

Bacteria were grown to late log phase (OD_600 _= 1.0, approximately 10^9 ^cells per ml), pelleted by centrifugation at 3 000 × *g *for 5 minutes and re-suspended in 50 mM Tris buffer pH 7.0 at approximately 10^11 ^cells per ml. One ml of suspension was added to 0.5 ml fosmidomycin solution and 3.5 ml LB media for *E*. coli and *M. smegmatis *or Middlebrook 7H9/Tw/OADC medium for *M. tuberculosis *and incubated at 37°C for 30 or 60 min with shaking at 100 rpm (*E. coli*, *M. smegmatis*) or standing (*M. tuberculosis*). Cells were pelleted by centrifugation at 3 000 × *g *for 5 minutes, the supernatant was collected, filter sterilized through a 0.2 μm syringe filter and stored on ice. Cells were washed twice with 5 ml of 50 mM Tris pH 7.0, re-suspended in 1 ml of 10 mM Tris pH 7.0, transferred to a Fast Prep Lysing Matrix B tube (QBiogene) and incubated on ice for 5 min. Cell-free extracts were prepared using the Fast Prep at speed 4 for 30 seconds (QBiogene). Samples were incubated on ice for 5 min, cell debris pelleted, the cleared lysate recovered, filter sterilized and stored on ice.

### Fosmidomycin quantitation

Fosmidomycin was measured using a microbiological assay [34]. 100 ml of cell-free extracts or supernatants (and serial dilutions) were applied to a sterile 14 mm filter paper disc (Whatman). The discs were allowed to dry at room temperature for 15 minutes before transferral onto individual 90 mm agar plates containing 0.5% v/v of an *E. coli *overnight culture. Plates were incubated for 20 hours at 37°C and the diameter of the zone of bacterial growth inhibition was measured. Discs with known concentrations of fosmidomycin were used to make a standard curve, to calculate the concentration of fosmidomycin in the test solutions. The concentration of fosmidomycin in the samples was then calculated from the standard curve.

## Authors' contributions

TP conceived of the study, and participated in its design and coordination and drafted the manuscript. ACB conducted the experimental work, participated in its design and drafted the manuscript. All authors read and approved the final manuscript.

## Supplementary Material

Additional file 1 **Schematic representation of the bioassay used to quantify fosmidomycin**. Cells were exposed to fosmidomycin; cell-free extracts or culture filtrates were prepared and applied to sterile paper discs. The discs were aseptically placed on media containing *E. coli*, as a test organism. The diameter of the zone of growth inhibition was measured following incubation. Using known amounts of fosmidomycin a standard curve of growth inhibition was constructed and used to quantify the fosmidomycin in the samples. Click here for file
