# Supplementary figures and images for: Dxr is essential in Mycobacterium tuberculosis and fosmidomycin resistance is due to a lack of uptake
Source: BMC Microbiol. 2008 May 20;8:78. doi: 10.1186/1471-2180-8-78 (PMC2409342; doi:10.1186/1471-2180-8-78)

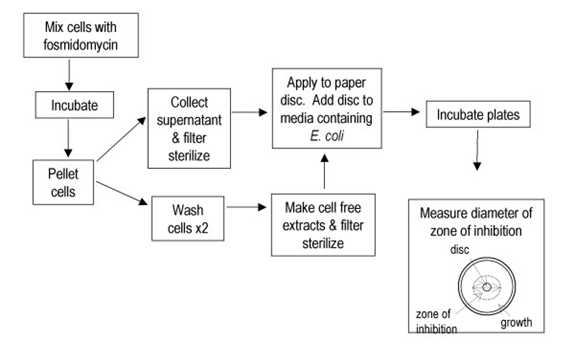

Supplement: Additional file 1 — Schematic representation of the bioassay used to quantify fosmidomycin. Cells were exposed to fosmidomycin; cell-free extracts or culture filtrates were prepared and applied to sterile paper discs. The discs were aseptically placed on media containing E. coli, as a test organism. The diameter of the zone of growth inhibition was measured following incubation. Using known amounts of fosmidomycin a standard curve of growth inhibition was constructed and used to quantify the fosmidomycin in the samples. [file 1471-2180-8-78-S1.tiff]
